# Characterization of an Oleaginous Unicellular Green Microalga, *Lobosphaera incisa* (Reisigl, 1964) Strain K-1, Isolated From a Tidal Flat in the Yellow Sea, Republic of Korea

**DOI:** 10.3389/fmicb.2018.02159

**Published:** 2018-09-10

**Authors:** Seungki Lee, Se Ra Lim, Dae Gwin Jeong, Ji Hyung Kim

**Affiliations:** ^1^Biological and Genetic Resources Assessment Division, National Institute of Biological Resources, Incheon, South Korea; ^2^Bio-Analytical Science Division, Korea University of Science and Technology, Daejeon, South Korea; ^3^Infectious Disease Research Center, Korea Research Institute of Bioscience and Biotechnology, Daejeon, South Korea

**Keywords:** microalgae, *Lobosphaera incisa* K-1, arachidonic acid, polyunsaturated fatty acids, biotechnological application

## Abstract

Microalgae are considered as sustainable resources for biofuel production. However, recently the focus on microalgal research has shifted toward the investigation of high-value metabolites for potential pharmaceutical and nutritional applications. Herein, we report the identification of a novel oleaginous green microalga isolated from the Yellow Sea in Korea. We also describe the morphological, molecular, and biochemical characteristics of this microalga. On the basis of microscopic and genetic analyses, the isolate was classified as *Lobosphaera incisa* (the strain was designated as K-1), and molecular phylogeny revealed that the isolate distinctly differed from the other known *L. incisa* strains. The microalga could be cultivated in various commercial culture media under a relatively broad range of pH and temperature conditions. We also did a rough and detailed estimation of the different cellular components in the microalga. The composition of arachidonic acid (C20:4ω6) in the lipids of *L. incisa* strain K-1 was relatively high, similar to that in other strains, however, the K-1 strain had higher proportions of the ω3 series of fatty acids (FAs), including α-linolenic acid (C18:3ω3) and eicosapentaenoic acid (C20:5ω3), highlighting its uniqueness and strong potential for biotechnological application. To the best of our knowledge, this is the first report on the isolation of *L. incisa* from Korea as well as from a marine environment; this novel strain might be useful for the production of high-value ω3 and ω6 polyunsaturated fatty acids (PUFAs).

## Introduction

Microalgae have been investigated as resources for alternative biofuel production. However, recently the research focus has changed toward using them as producers of high-value metabolites, such as antimicrobials, antioxidants, and polyunsaturated fatty acids (PUFAs), and nutritional supplements for humans and animals (Vanthoor-Koopmans et al., 2013). Microalgae contain high amounts of lipids, proteins, and carbohydrates, but one of the main bottlenecks in their use is that the composition of these compounds varies dramatically with the species and strains (Guedes et al., 2011). It is highly desirable to find appropriate microalgal species (or strains) with advantageous properties, which can be used for isolation of high-value metabolites.

Among the high-value metabolites present in microalgae, PUFAs have recently been under increased focus owing to their potential therapeutic uses and nutritional applications (Pulz and Gross, 2004). Fish and shellfish are among the major food sources of ω3 and ω6 PUFAs, however, several studies have shown that microalgae have competitive advantages over fish oils as nutritional sources of these PUFAs for humans because of the lack of unpleasant odor and reduced risk of heavy metal contamination (Guil-Guerrero et al., 2000). Moreover, microalgae have become one of the most important feed items in aquaculture owing to their innate capacity for synthesis of PUFAs (Tonon et al., 2002; Guedes et al., 2011). Therefore, microalgal PUFAs have a very promising biotechnological potential both as food and feed, and several microalgal PUFA products are already commercially available (Pulz and Gross, 2004).

*Lobosphaera incisa* (Reisigl, 1964) comb. nov. is a coccoid, unicellular microalga belonging to the class Trebouxiophyceae (Chlorophyta), which has been isolated from alpine soil, freshwater habitats, and lichen photobionts (Watanabe et al., 1996; Tourasse et al., 2015). This species was originally assigned to the genus *Myrmecia*, and then to *Parietochloris*, and was subsequently reclassified as *Lobosphaera* (Friedl, 1995; Watanabe et al., 1996; Karsten et al., 2005). This oleaginous microalga is the richest known plant source of arachidonic acid (AA, C20:4ω6), one of the pharmaceutically valuable ω6 long-chain PUFA, and has, therefore, been biologically and genetically investigated with enormous biotechnological interests (Bigogno et al., 2002a,b; Khozin-Goldberg et al., 2002; Abu-Ghosh et al., 2015; Tourasse et al., 2015, 2016).

Since 2010, we have been screening several microalgal and cyanobacterial strains with advantageous characteristics of their cellular components for biotechnological applications (Jeon et al., 2015; Kim et al., 2015a,b, 2017; Kim and Kang, 2016). Herein, we report the characterization of a unique indigenous strain of *L. incisa* isolated from a tidal flat in the Yellow Sea, Republic of Korea. We investigated the morphological, molecular, and biochemical characteristics of the isolated oleaginous microalga and evaluated its potential for biotechnological applications. To the best of our knowledge, this is the first report on the isolation of *L. incisa* from marine environment, and for the first time from Korea.

## Materials and Methods

### Isolation and Culture Conditions of Microalga

A unicellular coccoid green microalga was isolated from a sample of marine sediments collected from the tidal flat of Yellow Sea, Republic of Korea (36°53′35.0″N 126°19′40.8″E) in May, 2015. The water sample was serially diluted and incubated in 96-well plates at 25°C under 100 μmol photons/m^2^/s light intensity (12 h:12 h light:dark cycle) in BG-11 medium (pH 8.0, adjusted using NaOH or HCl) (Sigma-Aldrich, St. Louis, MO, United States). The unialgal cells were repeatedly streaked and cultivated on solid BG-11 medium plate (1.5% agar concentration, w/w) under the same conditions as mentioned above, until the pure isolate was obtained. All the subsequent analyses in this study were performed using axenic microalgal cells cultivated under the same conditions as mentioned above.

### Microscopy

The general morphology of the isolated microalga was investigated under a light microscope (LM) (Eclipse 80i; Nikon Co., Japan), and the size of cells was determined with an image analyzer (NIS-Elements BR 3.0; Nikon Co., Japan). For ultrastructural analysis, the microalgal cells were prepared as described previously (Kim et al., 2015a,b) and were examined at National Instrumentation Center for Environmental Management, Seoul National University, Seoul, Korea using a Field Emission SEM (AURIGA; Carl Zeiss, Germany) and TEM (JEM1010; JEOL Ltd., Japan), respectively.

### Molecular Identification and Phylogenetic Analysis

The microalgal genomic DNA was extracted using Plant DNA isolation reagent (Takara, Japan) according to the manufacturer’s instructions. The 18S rRNA gene and 18S–28S internal transcribed spacer (ITS) regions, including the D1/D2 region (ITS1-5.8S rRNA-ITS2-28S rRNA, hereinafter, referred to as the ITS region), were, respectively, amplified using universal NS1/NS3/NS8 primers and ITS1/ITS4/LR3R primers (Jeon et al., 2015). The amplified fragments were sequenced at Macrogen Inc. (Seoul, Republic of Korea). The sequences of the amplified 18S rRNA and ITS region were compared against the GenBank database using a BlastN search and were aligned with the corresponding sequences from other relatives of the phylum Chlorophyta using Clustal X (version 1.83) (Thompson et al., 1997) and BioEdit Sequence Alignment Editor (version 7.1.0.3) (Hall, 1999). The phylogenetic analysis of the microalgal 18S rRNA gene was conducted using the neighbor-joining (NJ) tree with Jukes-Cantor distance matrices and the maximum-likelihood (ML) method using the HKY model, suggested by jModelTest ver. 0.1.1 (Posada, 2008). The ITS region was phylogenetically analyzed using the NJ and maximum parsimony (MP) methods. The phylogenetic trees were reconstructed using MEGA ver. 7.0 (Kumar et al., 2016), and the reliability of trees was assessed by performing 1,000 bootstrap replicates. The nomenclature followed the Algae Base^1^ (Guiry and Guiry, 2018).

### Growth Characteristics

The growth of the isolated microalga in BG-11 medium was preliminarily compared to that in the other commercially available algal culture media, including Tris-acetate phosphate (TAP) medium (Thermo Fisher Scientific, Waltham, MA, United States), Guillard’s (f/2) medium (Sigma-Aldrich, St. Louis, MO, United States), and modified Bold’s Basal medium (BBM) (Sigma-Aldrich, St. Louis, MO, United States). The cultures of *L. incisa* K-1 in the exponential phase were batch cultivated in aerated 5-L flasks for 14 days under the same culture conditions as described above. As observed in a previous study on *L. incisa* SAG 2468 (Khozin-Goldberg et al., 2002), our isolate had a tendency to form aggregates and was thus not amenable to direct enumeration of cells. Therefore, 20 mL of culture was filtered through GF/C filter paper (Whatman Ltd., Maidstone, United Kingdom) for the estimation of cell density, and the total biomass was determined by weighing the filter paper before and after overnight drying in an oven at 50°C. The biomass of the isolated microalga was calculated by dividing the difference between the dry weights at the start and end of the experiment by the duration in days. Based on these results, the growth characteristics of the microalgal isolate in BG-11 medium were determined at a range of pH, salinity, and temperature conditions, with pH ranging from 3 to 11 (adjusted using NaOH or HCl), salinity varying from 0 to 50 psu (adjusted using NaCl), and temperature ranging from 10 to 35°C under the same conditions as mentioned above, excluding the variants.

### Analysis of Cellular Components

The proportions of the crude major components (ash, carbohydrate, lipid, moisture, and protein) in lyophilized microalgal cells cultivated in aerated 5L flasks (BG-11 medium, pH 8.0) at 25°C under 100 μmol photons/m^2^/s light intensity (12 h:12 h light:dark cycle) for 14 days were determined according to the official methods of the Association of Official Analytical Chemists (AOAC) (AOAC, 2006). The concentrations of amino acids, monosaccharides, and fatty acids (FAs) in the cells were determined as previously described (Kim et al., 2015a).

### Statistical Analysis

Statistical differences in all the experiments were determined using Student’s *t*-test. A *p*-value of less than 0.01 was accepted as statistically significant. The SPSS statistical software package version 13.0 (SPSS, Inc., Chicago, IL, United States) was used for all the analyses.

### Accession Numbers of Nucleotide Sequences and Strain Deposition

The sequences of 18S rRNA and ITS region of *L. incisa* K-1 were deposited in GenBank under the accession numbers, KT119888 and KT119889, respectively. A living axenic culture of *L. incisa* K-1 was deposited in the Korean Collection for Type Cultures (KCTC) at the Korean Research Institute of Bioscience and Biotechnology under the accession number KCTC 12888BP.

## Results and Discussion

### Morphology and Ultrastructure

**FIGURE 1:**
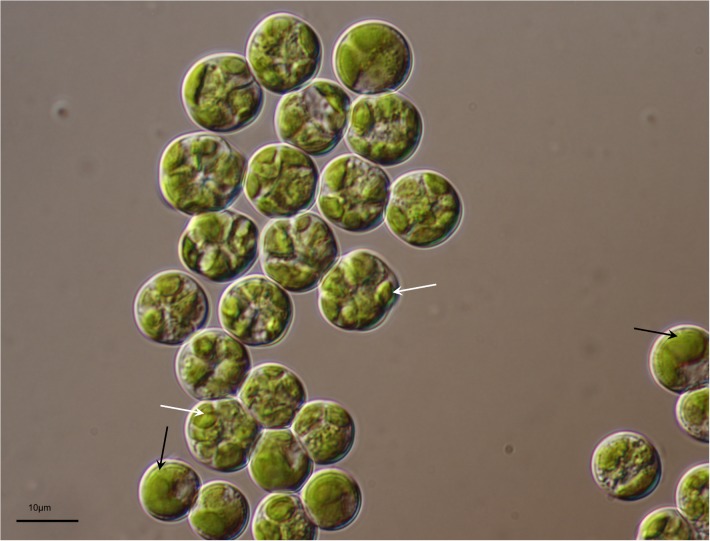
Light micrograph of *Lobosphaera incisa* K-1. Distinctive parietal and incised chloroplast (black arrow), and autospores (white arrow) are visible in the vegetative and mature cells, respectively.

**FIGURE 2:**
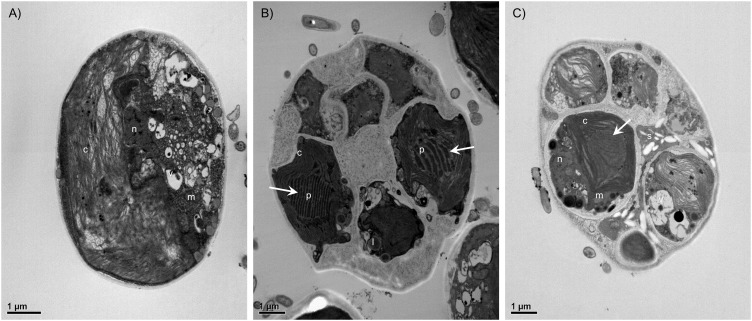
Transmission electron micrographs of *Lobosphaera incisa* K-1 showing the ultrastructure of vegetative cells **(A)** and cells during the asexual reproduction stage **(B,C)**. The parietal chloroplast distinctively contained pyrenoids, traversed by many parallel thylakoid membranes (white arrow). *c*, Chloroplast, *p*, Pyrenoid, *m*, Mitochondria, *s*, Starch granule, *l*, Lipid droplet, *n*, Nucleus, *v*, Vacuole.

The LM and SEM examinations of the culture revealed the presence of non-attaching and non-motile, spherical, or ovoid (9–13 μm in diameter), vegetative cells, mainly without zoospore production. The vegetative cells had parietal and incised chloroplasts with morphological variations, and the thickness of cell wall was almost even in the spherical cells. The mature cells were uninucleated and 4–32 autospores were released by rupture of the mother cell wall (**Figure 1**). The ultrastructure of the microalgal cells was further investigated using TEM, and several cellular organs, such as nuclei, chloroplasts, pyrenoids, mitochondria, starch granules, and lipid droplets were observed (**Figures 2A–C**). The parietal chloroplast found in the isolated microalga distinctively contained pyrenoids, traversed by many parallel thylakoid membranes; the presence of pyrenoids, which are considered to be the key feature in the taxonomical identification of *L. incisa* (Watanabe et al., 1996), suggested that the newly isolated microalga could be classified into the genus *Lobosphaera*. The accumulation of starch granules and lipid droplets was rarely observed in the cells.

### Molecular Identification and Taxonomic Characterization

The sequences of 18S rRNA (1,627 bp) and ITS region (1,157 bp) of the isolate were analyzed using those of other microalgal species available in the GenBank database. A BLASTN search using the obtained 18S rRNA sequence as a query revealed that the closest related species were *L. tirolensis* ASIB S234 (GenBank accession no. AB006051), which is the type species in the genus *Lobosphaera*, and *L. incisa* SAG 2466 (GenBank accession no. KM020046), with 99.7% sequence identity for both. Although the sequences of the isolate K-1 were also very similar to those of *P. ovoidea* ACKU 177-03 (GenBank accession no. EU878374), with 99.7% sequence identity, the micoalga showed only 97.0% sequence identity to the type species of the genus *Parietochloris*, *P. alveolaris* UTEX 836 (GenBank accession no. EU878373). In addition, comparison of the sequence of ITS region of the isolate and those of Chlorophyta present in GenBank revealed the highest sequence identity (93.1%) with *L. incisa* SAG 2466 (GenBank accession no. KM020046), followed by 89.4% sequence identity with *Chlorella ellipsoidea* IAM C-87 (GenBank accession no. D17810) and 83.5% with *C. sorokiniana* UTEX1665 (GenBank accession no. KJ676113). Based on these results, the newly isolated microalga was classified into the genus *Lobosphaera*.

**FIGURE 3:**
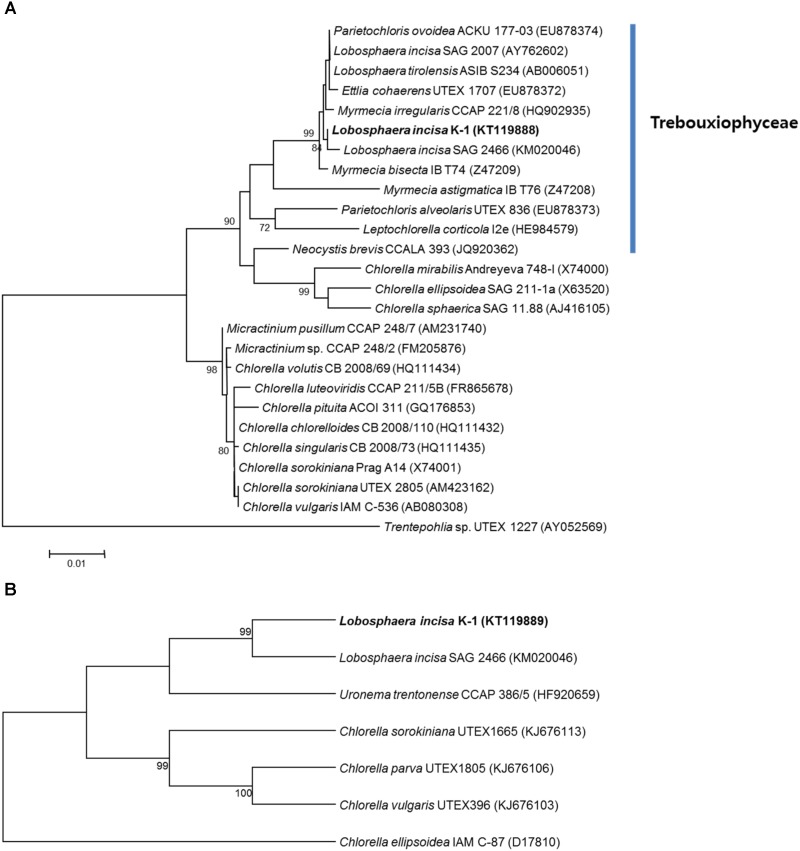
Maximum-likelihood tree reconstructed using a data set of 26 18S rRNAs derived from the genus *Lobosphaera* and its relative species in Chlorophyta **(A)**. The genera *Ettlia*, *Myrmecia*, *Parietochloris*, and *Lobosphaera* could not be clearly separated from each other, and our isolate (KT119888) formed different lineages with *L. incisa* SAG 2466 (KM020046). *Trentepohlia* sp. UTEX 1227 (AY052569) was used as an outgroup. The scale bar represents 0.01 nucleotide substitutions per site. Maximum-parsimony tree reconstructed using a data set of seven internal transcribed spacer (ITS) regions derived from the genus *Lobosphaera* and its available relatives **(B)**. Numbers at the branches indicate bootstrapping values obtained with 1,000 replicates; only bootstrap values >70% are indicated, respectively.

Although several recent molecular phylogenetic analyses of the members of Trebouxiophyceae revealed ambiguous taxonomic position of the genus *Lobosphaera* (Henley et al., 2004; Karsten et al., 2005), detailed phylogenetic analyses were conducted using a data set of 26 18S rRNA and seven ITS region sequences derived from the unicellular coccoid, Chlorophyta, to determine the taxonomic position of the isolate. The phylogenetic tree constructed using the ML method also revealed that the microalgal strains classified in the genera *Ettlia*, *Myrmecia*, *Parietochloris*, and *Lobosphaera*, available in GenBank, could not be clearly separated from each other, however, the isolate identified by us clearly formed different lineages with *L. incisa* SAG 2466 among the other related microalgal strains (**Figure 3A**). Similar results were obtained based on the phylogenetic trees constructed using the NJ method (data not shown). The phylogenetic analysis of the ITS region using the MP method also clustered the microalgal isolate together with the *L. incisa* SAG 2466 strain, but the isolate formed a separate lineage, showing divergence (**Figure 3B**). In addition, a similar tree topology was obtained when the NJ method was used (data not shown). Because of the absence of 18S and ITS sequences of the most thoroughly investigated *L. incisa*, strain SAG 2468, in GenBank, we were not able to directly compare the isolate to all the other available *L. incisa* strains. However, our microalgal isolate showed distinct differences in the ITS region and in the source of isolation when compared to the other available *L. incisa* strains. Based on these results, the newly isolated microalga was finally classified as a unique strain (designated as K-1) of *L. incisa*. We further investigated its growth and biochemical characteristics to evaluate the potential for its use as a novel indigenous bioresource.

### Growth Characteristics

**FIGURE 4:**
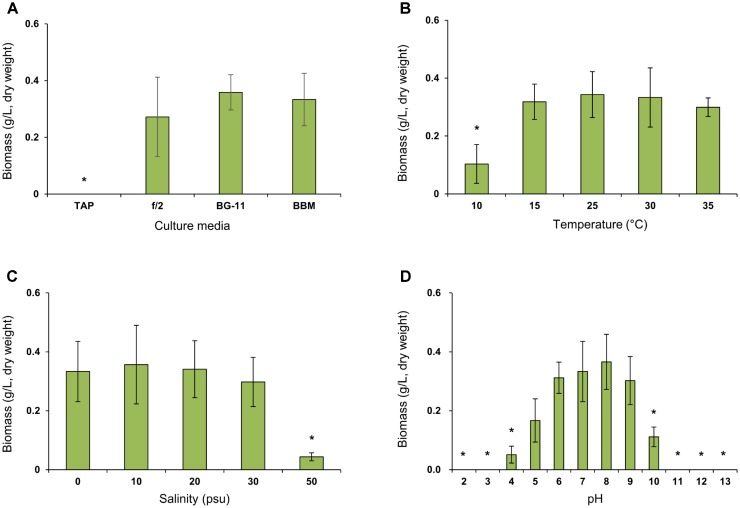
Comparison of biomass concentration (g/L, dry weight) of *Lobosphaera incisa* K-1 in different culture media **(A)**, temperature **(B)**, salinity **(C)**, and pH **(D)** conditions. Asterisk indicates the groups that showed significant differences (*p* < 0.01) to our initial isolation and culture condition of *L. incisa* K-1 using BG-11 medium (pH 8.0 and 0 psu) at 25°C. The results are shown as means ± standard deviations from triplicate experiments.

*Lobosphaera incisa* SAG 2468 is routinely cultivated in BG-11 medium (Bigogno et al., 2002a,b; Khozin-Goldberg et al., 2002; Abu-Ghosh et al., 2015; Tourasse et al., 2016). Therefore, we compared the biomass production of the K-1 strain in BG-11 and other commercially available algal culture media, for future applications (**Figure 4A**). The growth of *L. incisa* K-1 in BBM was comparable to that in BG-11, without any noticeable differences, however, no growth was observed on TAP medium. The total biomass production was slightly decreased in f/2 medium and strongly aggregated groups of entangled cells were observed to settle to the bottom of the culture flask. Based on these results, BG-11 medium was selected for the production of microalgal biomass to investigate the cellular biochemical components in this study, and its growth characteristics in the culture medium were determined under different pH, salinity, and temperature conditions (**Figures 4B–D**). The microalga could be cultured at temperatures ranging from 10 to 35°C in BG-11 medium, with the most rapid growth observed between 25 and 30°C. Moreover, the isolate could be cultured under a wide range of salinity (ranging from 0 to 50 psu) and pH (ranging from 4 to 10), however, optimal growth was recorded at salinities ranging from 0 to 30 psu and at pH ranging from 6 to 9.

### Cellular Components

To evaluate the potential of *L. incisa* K-1 as a microalgal bioresource, the proportions of cellular components were determined. The overall composition of crude cellular components in *L. incisa* K-1 was similar to that reported in other green microalgae belonging to the class Chlorophyceae (Becker, 2007). In the lyophilized microalgal cells, the ash, carbohydrate, lipid, and protein contents were determined to be 6.2 ± 0.0%, 28.1 ± 0.6%, 20.4 ± 0.3%, and 43.5 ± 0.3%, respectively. Several microalgal species (mainly *Chlorella*) are currently used as dietary supplements, and their cellular protein content is considered to be the major factor determining the nutritional value of microalgae (Spolaore et al., 2006). In this study, *L. incisa* K-1 was also found to contain a relatively high proportion of crude protein, making it a potential candidate for use as a dietary supplement similar to other commercially available microalgal species (Spolaore et al., 2006).

We also determined the composition of amino acids, monosaccharides, and FAs in *L. incisa* K-1. Because of the absence of data on the composition of amino acids and monosaccharides in the most thoroughly investigated *L. incisa* strain SAG 2468, we could not directly compare the compositions in the isolate to those in the other available *L. incisa* strains. Of the total amino acids present in *L. incisa* K-1, 42.3 ± 0.6% were the essential amino acids. The dominant amino acids were glutamic acid (12.6 ± 0.1%), aspartic acid (10.6 ± 0.0%), and alanine (10.9 ± 0.0%), whereas methionine, cysteine, and tryptophan were not detected (**Table 1**). Although most of the algal (including microalgal) species are known to have limited amounts of specific amino acids, such as cysteine, tryptophan, and lysine (Bleakley and Hayes, 2017), *L. incisa* K-1 distinctively contained relatively high amount of lysine (8.0 ± 0.3%) (Becker, 2007). The monosaccharide composition of the polysaccharides in *L. incisa* K-1 was also analyzed in this study. D-galactose (32.2 ± 1.5%), D-glucose (25.1 ± 2.1%), and D-mannose (19.4 ± 1.0%) were identified to be the dominant monosaccharides present in this strain (**Table 2**). According to previous reports (Brown and Jeffrey, 1992; Brown et al., 1997), the predominant monosaccharide in most Chlorophytes is glucose, however, our isolate contained galactose as its predominant monosaccharide. Additionally, the contents of mannose and xylose were relatively higher than in other Chlorophytes (Brown et al., 1997).

**Table 1:** Compositions (%) of amino acids in lyophilized *L. incisa* K-1^a^.

	Asp	Thr^b^	Ser	Glu	Gly	Ala	Cys2	Val^b^	Met^b^	Ile^b^	Leu^b^	Tyr	Phe^b^	His^b^	Lys^b^	NH_4_	Arg	Pro	Trp	Total
Contents (mg/g)	5.6 ± 0.9	2.4 ± 0.4	2.9 ± 0.5	6.7 ± 1.2	2.7 ± 0.5	5.8 ± 1.0	–^c^	3.5 ± 0.6	–	2.5 ± 0.4	5.2 ± 1.0	0.6 ± 0.1	2.6 ± 0.4	2.1 ± 0.3	4.3 ± 0.8	1.1 ± 0.2	3.6 ± 0.6	1.7 ± 0.2	–	53.4 ± 8.8
Compositions (%)	10.6 ± 0.0	4.5 ± 0.0	5.4 ± 0.0	12.6 ± 0.1	5.1 ± 0.1	10.9 ± 0.0	–	6.6 ± 0.0	–	4.7 ± 0.1	9.7 ± 0.3	1.2 ± 0.1	4.8 ± 0.0	4.0 ± 0.0	8.0 ± 0.3	2.0 ± 0.0	6.7 ± 0.0	3.2 ± 0.9	–	100

*^a^Asp, aspartic-acid; Thr, threonine; Ser, serine; Glu, glutamic acid; Gly, glycine; Ala, alanine; Cys2, cystine-cystine dimer; Val, valine; Met, methionine; Ile, isoleucine; Leu, leucine; Tyr, tyrosine; Phe, phenylalanine; His, histidine; Lys, lysine; NH_4_, Ammonia; Arg, arginine; Pro, prolin; Trp, tryptophan. ^b^Essential amino acids. ^c^Not detected.*

**Table 2:** Compositions (%) of monosaccharides in lyophilized *L. incisa* K-1.

Monosaccarides (%)	Contents (mg/g)	Compositions (%)
L(−) fucose	5.4 ± 0.3	3.3 ± 0.6
L(−) rhamnose	11.8 ± 2.0	7.1 ± 0.7
D(+) arabinose	1.3 ± 0.1	0.8 ± 0.2
D(+) galactose	53.2 ± 3.2	32.2 ± 1.5
D(+) glucose	41.2 ± 2.0	25.1 ± 2.1
D(+) mannose	32.1 ± 0.9	19.4 ± 1.0
D(+) xylose	20.0 ± 1.9	12.1 ± 0.3
Total	164.9 ± 10.5	100.0

The FA content and composition of the isolated microalga were compared with those of *L. incisa* strain SAG 2468. The total FA content of *L. incisa* K-1 cultured for 14 days was estimated to be 16.6 ± 1.9% of the total biomass (dry weight), which was very similar to that of the strain SAG 2468 (Khozin-Goldberg et al., 2002). We also assessed the FA composition in *L. incisa* K-1 in detail and compared it with the composition in the strain SAG 2468 (Bigogno et al., 2002a; **Table 3**).

**Table 3:** Comparison of fatty acid compositions (%) in lyophilized *L. incisa* K-1 and other microalgae belonging to the genus *Lobosphaera*.

Fatty acids	*L. incisa* K-1	*L. incisa* SAG 2468^a^
C14:0	0.1 ± 0.0	–^b^
C15:0	0.9 ± 0.2	–
C16:0	17.0 ± 0.3	10.1
C16:1ω11	–	1.8
C16:1ω7	–	tr
C16:1ω9	0.7 ± 0.0	–
C16:2ω6	2.4 ± 0.2	1.3
C16:3ω3	4.2 ± 0.4	0.9
C16:4ω3	0.3 ± 0.1	–
C18:0	1.3 ± 0.4	2.5
C18:1ω7	–	4.2
C18:1ω9	10.6 ± 0.7	12.2
C18:2ω6	19.4 ± 0.6	17.2
C18:3ω3	22.9 ± 0.4	2.0
C18:3ω6	1.2 ± 0.4	0.8
C18:4ω3	0.5 ± 0.1	–
C20:1ω9	0.2 ± 0.1	–
C20:3ω6	–	1.0
C20:4ω6	15.3 ± 0.3	42.5
C20:5ω3	2.3 ± 0.4	0.7
C24:0	0.2 ± 0.1	–
C24:1ω9	0.8 ± 0.2	–
Σ SFAs	19.4 ± 0.7	12.6
Σ MUFAs	12.2 ± 1.0	18.2
Σ PUFAs	68.1 ± 0.1	66.4
Total	99.7	97.2

*^a^Bigogno et al. (2002a). ^b^Not detected.*

The FAs in our isolate comprised of saturated fatty acids (SFAs) (19.4 ± 0.7%), monounsaturated fatty acids (MUFAs) (12.2 ± 1.0%), and PUFAs (68.1 ± 0.1%), and the overall FA profile of our isolate was similar to that of the strain SAG 2468 (Bigogno et al., 2002a). The lipids in the strain K-1 mainly contained C18 and C20 FAs, such as C18:1ω9 (elaidic acid, 10.6 ± 0.7%), C18:2ω6 (linoleic acid, LA, 19.4 ± 0.6%), and C20:4ω6 (AA, 15.3 ± 0.3%). However, the composition of FAs in *L. incisa* K-1 distinctly differed from that in the strain SAG 2468 as follows: first, *L. incisa* K-1 contained a high proportion of C18:3ω3 (α-linolenic acid, ALA, 22.9 ± 0.4%), the precursor of the ω3 series of FAs, which cannot be produced in human body and is, thus, essential in the human diet (Wall et al., 2010). This indicates that the strain K-1 produces ω3-desaturase that can synthesize ALA from LA (Guedes et al., 2011), unlike in the known *L. incisa* strain SAG2468; second, although the actual composition was low, our isolate could produce C20:5ω3 (eicosapentaenoic acid, EPA, 2.3 ± 0.4%) without the presence of its precursor, C20:4ω3 (eicosatetraenoic acid), suggesting that *L. incisa* K-1 might have Δ*17-desaturase* that can directly synthesize EPA from AA (Guedes et al., 2011). These biochemical characteristics also supported the uniqueness of the newly isolated *L. incisa* K-1 from the Yellow Sea, Republic of Korea.

Based on these results, *L. incisa* K-1 potentially has strong advantages as a microalgal bioreactor for the production of high-value ω3 and ω6 long-chain PUFAs (especially AA and EPA), which are widely acclaimed for promoting human health (Tapiero et al., 2002). Moreover, because the antimicrobial (Falaise et al., 2016) and antiviral activities (Kohn et al., 1980; Thormar et al., 1987) of long-chain PUFAs have been verified, the newly isolated microalga, enriched in long-chain PUFAs, can also have a broad spectrum of applications, such as in pharmaceuticals, cosmetics, and environmental restoration. Further studies are currently in progress to determine the optimal conditions for the culture of *L. incisa* K-1 to increase the productivity of microalgal biomass and the accumulation of long-chain PUFAs, especially AA and EPA, for practical use.

## Conclusion

The focus of microalgal research has recently changed toward investigating the potential of microalgae to produce high-value metabolites other than biofuel. The oleaginous green microalga, *L. incisa*, has been relatively well investigated because of its rich composition of AA of plant origin. In this report, we describe the isolation of a unique strain (designated as K-1) of microalga, which has strong potential for biotechnological application. The isolate was genetically different from the other available *L. incisa* strains, and could produce several valuable PUFAs besides AA. To the best of our knowledge, this is the first report of *L. incisa* isolated from Korea, as well as from a marine environment. This microalga might be useful for the production of high-value ω3 and ω6 PUFAs.

## Author Contributions

SL and SRL performed the experiments and prepared the draft manuscript. DJ contributed to the manuscript discussion and revision. JK contributed to the experimental design, discussion of results, manuscript revision, and overall support of this study.

## Conflict of Interest Statement

The authors declare that the research was conducted in the absence of any commercial or financial relationships that could be construed as a potential conflict of interest.

## Footnotes

1 http://www.algaebase.org
